# Living through the COVID-19 Pandemic: Impact and Lessons on Dietary Behavior and Physical Well-Being

**DOI:** 10.3390/ijerph19020642

**Published:** 2022-01-06

**Authors:** Shameena Gill, Alia Maisara Adenan, Adli Ali, Noor Akmal Shareela Ismail

**Affiliations:** 1 Department of Biochemistry, Faculty of Medicine, Universiti Kebangsaan Malaysia, Jalan Yaacob Latif, Cheras, Kuala Lumpur 56000, Malaysia; shameenagill@gmail.com (S.G.); aliamaisara18@gmail.com (A.M.A.); 2 Department of Pediatrics, Faculty of Medicine, Universiti Kebangsaan Malaysia, Jalan Yaacob Latif, Cheras, Kuala Lumpur 56000, Malaysia; adli.ali@ppukm.ukm.edu.my

**Keywords:** COVID-19, lockdown, dietary behavior, nutrition, physical activity

## Abstract

The aim of this review is to highlight the spectrum on which human behavior has been affected by blanket restriction measures and on a wider scale, the COVID-19 pandemic. Some of the human behaviors that have been impacted by the COVID-19 lockdown are dietary behavior and nutrition, food options and food delivery usage, physical activity and sedentary behaviors. This is important in planning effective public health strategies with minimal detriment to all subsets of society as well as improving the distribution of government aid to populations that are more severely affected. Our main purpose is to present the literature from a rapidly growing pool of scientific research to hopefully enable a better and more comprehensive understanding of the effects of this pandemic and the lessons learnt from the accompanying restrictions, as well as policy recommendations that can be made in national pandemic responses in the future.

## 1. Introduction

### 1.1. COVID-19—A Global Pandemic

The Coronavirus disease 2019 (COVID-19) is a severe acute respiratory syndrome which is caused by the novel coronavirus SARS-CoV-2. It first appeared in China in late 2019 [1] and presented as a cluster of patients who were afflicted with pneumonia of an unknown origin. These clusters were then traced back to one common location—a seafood wet market in Wuhan, Hubei province, China [2]. By late January 2020, in view of the alarming spread of the virus to different continents, the World Health Organization (WHO) had classified it as a Public Health Emergency of International Concern [3]. In view of increasing outbreaks around the globe, it was then characterized as a global pandemic on the 11 March 2020 [3]. Many governments around the world implemented a ‘lockdown’ strategy to manage and attempt to lower the number of new COVID-19 infections. There was a blanket prohibition on all mass gatherings, regardless of their nature. Numerous other restrictions were also imposed, including but not limited to the closure of public spaces such as restaurants, playgrounds and parks, and shopping centers, as well as an implementation of distance learning, mandatory quarantine for travelers entering the country, and limitations on the number of people that were allowed to go out from each household. Despite that, there have been 223,022,538 confirmed cases of COVID-19, which includes 4,602,882 deaths globally as reported by the World Health Organization (WHO) on 10 September 2021 at precisely 4:47 p.m. CEST [4].

**Figure 1:**
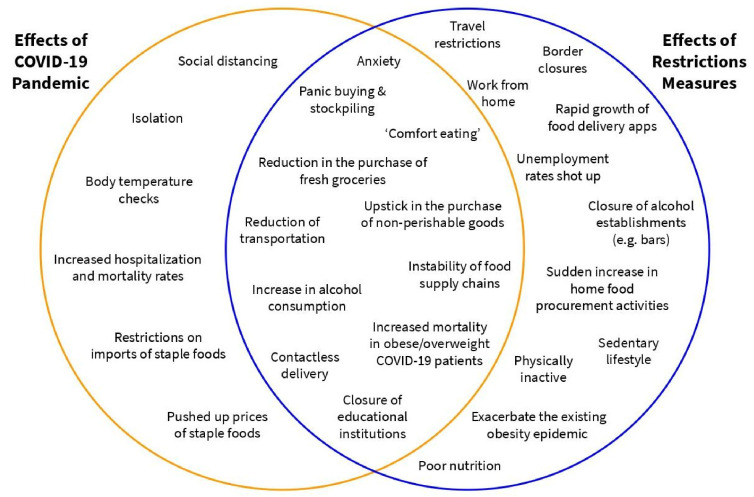
Effects of the COVID-19 Pandemic and Restriction Measures.

The comprehensive understanding of the effects of a lockdown is crucial in order to reduce the deleterious effects that may negatively impact control and prevention measures in tackling a pandemic. An apt example of this would be the phenomenon known as pandemic lockdown fatigue—a state of exhaustion or ‘burnout’ that is primarily caused by the far-reaching changes that COVID-19 has caused to normal daily lifestyles, especially in terms of people’s freedom to move around [5,6]. Prolonged lockdowns and restriction measures also bring with them a slew of long-term effects in various aspects socially, psychologically and economically (Figure 1).

### 1.2. Overview of the Current Paper

The main aim of this scoping review is to lay out an overview on the implications of COVID-19 restriction measures and its effects on human lifestyle based on changes in dietary behavior, nutrition, food options and food delivery usage, physical activity and sedentary behaviors. We focus on social, psychological and economic aspects of the aforementioned changes as a consequence to the COVID-19 pandemic outbreak. This paper provides a scoping overview of the literature on COVID-19 with the goal of identifying mutually inclusive effects of both the pandemic and its restrictive measures (Figure 1). Finally, we propose several policies based on lessons learnt from the past few years that may influence the future as we slowly progress from COVID-19 being a pandemic to an endemic, with the aim of reducing detrimental health effects to human well-being and improving the quality of life socially, psychologically and economically.

## 2. Methodology of Current Literature

The following subsections will address the methodology used to produce the current literature based on the search strategy and the study selection on the effects of the COVID-19 pandemic and restriction measures.

### 2.1. Search Strategy

We have carefully followed the Preferred Reporting Items for Systematic Reviews and Meta-Analyses (PRISMA) guideline prior to writing this review. The keywords used in the literature search were “COVID-19”, “dietary behavior”, “nutrition”, “food options”, “food delivery options”, “physical activity” “sedentary behaviors’’ and several subthemes (Table 1) since the inception of COVID-19 starting from November 2019 until 20 October 2021. However, our initial search revealed the complexity of the multiple keywords and restricted our discussion upon different themes that we intended to examine; thus, a scoping review was decided to be a more appropriate approach to address the role of restriction measures towards various parts of human lives. The keywords searched are systematically filtered based on the content of discussion in the studies that are applicable with emphasis on the keywords “COVID-19” and “pandemic” for each subtheme (Table 1). “COVID-19” and its relevant keywords are significant in our search process to obtain recent information appropriate to this novel pandemic that can be used for discussion in this scoping review. As each subtheme title is included in the keywords of our search strategy, subsequent keywords are chosen based on information of importance acquired from relevant studies that would be necessary to use for content discussion on the effects of the COVID-19 pandemic and restriction measures.

### 2.2. Study Selection

This scoping review consists of the latest and relevant literature to address all subsections in this manuscript, particularly in abiding by a general PRISMA guideline, including identifying the relevant publications, selecting publications relevant to the multiple keywords pertaining to COVID-19 and critically appraising the publications without any bias. All study designs were considered, including but not limited to experiments, surveys, focus groups, narrative reviews, and systematic literature reviews. The inclusion criteria for this scoping review are that all papers reviewed were written in English and discussed the effect of COVID-19 on various aspects of daily life (dietary behavior, physical activity, and psychological effect, etc.) and as for its exclusion criteria, duplicate studies were rejected. All literature presented in this scoping review was obtained from Google Scholar, PubMed, NCBI, WHO, and other governmental agencies such as the Centre for Diseases as main information sources. However, older publications are also used to illustrate certain pre-established facts. Each of the following subsections was carefully discussed based on the above methodology of the current literature. PRISMA guidelines were followed where possible; however, given that the current research is a scoping review, these were largely not applicable.

**Table 1:** Search Strategy, Study Selection on the Effects of the COVID-19 Pandemic and Restriction Measures.

Theme	Subtheme	Keyword	Author	Articles	Country	n Sample	Type of Paper
Dietary Behavior	Food security	COVID-19, pandemic, food choices, food security, shopping, purchase, behavior, panic buying, consumer changes, food supply chain, agriculture, food, heart disease, diet, malnutrition, cardiovascular, respiratory, cancer, NCDs, policies, fresh food, fruits, vegetables, immune system, chronic disease, inflammation, global, economy, food crisis, food production, prices, crop, farmer, sales, disruptions, demand, income, trade, poverty, logistics	Bracale, R. et al.	Changes in food choice following restrictive measures due to COVID-19	Italy	10,769 stores	Longitudinal Observational Study
			Di Crosta et al.	Psychological factors and consumer behavior during the COVID-19 pandemic.	Italy	3833 participants	Cross-sectional Observational Study
			Aday, S. et al.	Impact of COVID-19 on the Food Supply Chain.	N/A	N/A	Narrative Review
			Branca, F. et al.	Transforming the food system to fight non-communicable diseases.	N/A	N/A	Editorial
			Glade M. J. et al.	Food, nutrition, and the prevention of cancer: a global perspective	Worldwide	N/A	Scoping Review
			He, F. et al.	Increased consumption of fruit and vegetables is related to a reduced risk of coronary heart disease: Meta-analysis of cohort studies.	United Kingdom	278,459 individuals	Systematic Review
			Luckstead, J. et al.	Labor issues in the food supply chain amid the COVID-19 pandemic	United States of America	1648 respondents	Cross-sectional Observational Study
			Bagatini, M. et al.	Immune system and chronic diseases.	Brazil	N/A	Editorial
			Alam, G. et al.	Impact of COVID-19 on vegetable supply chain and food security: Empirical evidence from Bangladesh	Bangladesh	120 respondents	Longitudinal Observational Study
			Pan, D. et al.	The influence of COVID-19 on agricultural economy and emergency mitigation measures in China: A text mining analysis.	China	337 WeChat articles, 490 Weibo articles ~750,000 words	Scoping Review
			Laborde, D. et al.	COVID-19 risks to Global Food Security	United States of America	N/A	Editorial
			Commitee on World Food Security (CFS) High Level Panel of Experts (HLPE)	Impacts of COVID-19 on food security and nutrition: developing effective policy responses to address the hunger and malnutrition pandemic.	Italy	N/A	Longitudinal Observational Study
			Laborde, D. et al.	Impacts of COVID-19 on Global Poverty, food security, and diets: Insights from Global Model Scenario Analysis	Worldwide	IFPRI’s global general equilibrium model	Cohort Observational Study
	HFP activities	home food procurement (HFP), gardening, home food procurement, COVID-19, pandemic, food insecurity, poverty, foraging, fruits, vegetables, healthy lifestyle, community gardens, social benefit, nutrition, food system, urban agriculture, organic food, community, ecosystem, home gardens, malnourishment, nutritional security	Niles, M. T. et al.	Home food procurement impacts food security and diet quality during COVID-19	United States of America	600 residents	Cross-sectional Observational Study
			van den Berg, A. E. et al.	Allotment Gardening and health: A comparative survey among allotment gardeners and their neighbors without an allotment	Netherlands	184 respondents (121 + 63 control)	Cross-sectional Observational Study
			Algert, S. J. et al.	Vegetable output and cost savings of community gardens in San Jose, California.	United States of America	83 gardeners	Longitudinal Observational Study
			Nova, P. et al.	Urban Organic Community Gardening to promote environmental sustainability practices and increase fruit, vegetables and organic food consumption.	Portugal	115 city dwellers	Longitudinal Observational Study
			Lampert, T. et al.	Evidence on the contribution of community gardens to promote physical and mental health and well-being of non-institutionalized individuals: A systematic review	Worldwide	8 studies	Systematic Review
			Lal, R. et al.	Home Gardening and urban agriculture for advancing food and nutritional security in response to the COVID-19 pandemic.	United States of America	N/A	Editorial
	Alcohol Consumption	alcohol consumption, pandemic, e-commerce, alcohol sales, COVID-19, reward pathway, neural pathways, addiction	Scarpetta, S. et al. (Organisation for Economic Co-operation and Develeopment; OECD)	The effect of COVID-19 on alcohol consumption, and policy responses to prevent harmful alcohol consumption	Worldwide	N/A	Prospective Study
			Koob, G. et al.	Stress, dysregulation of drug reward pathways, and the transition to drug dependence.	United States of America	N/A	Retrospective Study
			Steffen, J. et al.	Altered alcohol consumption during COVID-19 pandemic lockdown.	Germany	2067 participants	Cross-sectional Observational Study
Nutrition	Social behavior	social behavior, anxiety, COVID-19, pandemic, psychological impact, depression, mental health, comfort food, neurophysiological mechanism, intrinsic reward mechanism, hyperpalatable food, obesity, inflammation, visceral weight gain, cardiovascular, adipose tissue, complications, calories, nutrient density, hunger, insulin resistance, cancer, body mass index (BMI), intensive care unit, SARS-Cov-2	Wang, C. et al.	Immediate psychological responses and associated factors during the initial stage of the 2019 coronavirus disease (COVID-19) epidemic among the general population in China	China	1210 respondents	Cross-sectional Observational Study
			Weltens, N. et al.	Where is the comfort in Comfort Foods? mechanisms linking fat signaling, reward, and emotion	N/A	N/A	Narrative Review
			Fazzino, T. L. et al.	Hyper-palatable foods: Development of a quantitative definition and application to the US Food System Database	United States of America	75 HPF descriptors	Systematic Review
			Hauner, H.	Secretory factors from human adipose tissue and their functional role: Proceedings of the nutrition society	N/A	N/A	Editorial
			Fuhrman, J. et al.	Changing perceptions of hunger on a high nutrient density diet	United States of America	768 participants	Cross-sectional Observational Study
			Shuster, A. et al.	The clinical importance of visceral adiposity: A critical review of methods for visceral adipose tissue analysis.	Worldwide	N/A	Narrative Review
			Mohammad, S. et al.	Obesity and COVID-19: What makes obese host so vulnerable?	Worldwide	N/A	Narrative Review
			Simonnet, A. et al.	High prevalence of obesity in severe acute respiratory syndrome coronavirus-2 (SARS-CoV-2) requiring invasive mechanical ventilation.	France	124 patients	Retrospective Study
			Ouchi, N. et al.	Adipokines in inflammation and metabolic disease.	Worldwide	N/A	Narrative Review
	Mediterranean diet	Mediterranean diet, health benefits, COVID-19, adipose tissue, inflammation, olive oil, polyphenols, cardiovascular health, anti-inflammatory, thrombosis, mast cells, polyphenols, immunomodulation, cytokines, diabetes, chronic disease, atherosclerosis, food choice, mortality, financial cost, hospitalisation	Bach-Faig, A. et al.	Mediterranean diet pyramid today. science and cultural updates	Mediterranean region	N/A	Prospective Study
			Angelidi A. M. et al.	Mediterranean diet as a nutritional approach for COVID-19.	N/A	N/A	Editorial
			Theoharides, T. C. et al.	Coronavirus 2019, Microthromboses, and platelet activating factor	N/A	N/A	Editorial
			Shakoor, H. et al.	Immunomodulatory effects of dietary polyphenols.	Worldwide	167 papers	Systematic Review
			Ding, S. et al.	Regulation of immune function by polyphenols	N/A	N/A	Narrative Review
			Maiorino, M. I. et al.	Mediterranean diet and COVID-19: Hypothesizing potential benefits in people with diabetes	N/A	N/A	Editorial
			Casas, R. et al.	The immune protective effect of the Mediterranean diet against chronic low-grade inflammatory diseases	N/A	N/A	Narrative Review
			Greene, M. W. et al.	Negative association between Mediterranean diet adherence and COVID-19 cases and related deaths in Spain and 23 OECD countries: An ecological study	OECD countries	24 countries	Cohort Observational Study
			Lampropoulos, C. E. et al.	Effects of Mediterranean diet on hospital length of stay, medical expenses, and mortality in elderly, hospitalized patients: A 2-year observational study	Greece	183 patients	Longitudinal Observational Study
			Mohajeri, M. et al.	The food choice determinants and adherence to Mediterranean diet in Iranian adults before and during COVID-19 lockdown: Population-based study	Iran	2540 adults	Cross-sectional Observational Study
	Intermittent Fasting	glucose, glycolysis, COVID-19, cytokine storm, intermittent fasting, metabolism, metabolic pathways, respiratory virus, SARS-CoV-2, diabetes, pandemic	Wang, Q. et al.	O-glcnac transferase promotes influenza A virus–induced cytokine storm by targeting interferon regulatory factor–5.	N/A	N/A	Experimental Study
			Yang, L. et al.	The signal pathways and treatment of cytokine storm in COVID-19	N/A	N/A	Scoping Review
			Codo, A. C. et al.	Elevated glucose levels favor SARS-CoV-2 infection and monocyte response through a HIF-1α/glycolysis-dependent axis	N/A	N/A	Experimental Study
			Lee, J. H. et al.	Intermittent fasting: Physiological implications on outcomes in mice and men	N/A	N/A	Narrative Review
			Albosta, M. et al.	Intermittent fasting: Is there a role in the treatment of diabetes? A review of the literature and guide for Primary Care Physicians.	United States of America	17 articles	Narrative Review
			Ealey, K. N. et al.	COVID-19 and obesity: Fighting two pandemics with intermittent fasting	N/A	N/A	Narrative Review
Food Options	Food Delivery	food delivery, food delivery apps, FDAs, online transactions, COVID-19, online food purchase lockdown, economic impacts, takeaways, diet quality, food choice, obesity, weight gain, eye level, psychology, supermarket purchase, nutrition, inflammation	Li, C. et al.	Review of online food delivery platforms and their impacts on sustainability	N/A	N/A	Narrative Review
			Muangmee, C. et al.	Factors determining the behavioral intention of using food delivery apps during COVID-19 pandemics	Thailand	402 respondents	Cross-sectional Observational Study
			Duda-Chodal, A et al.	COVID-19 pandemic and food: Present knowledge, risks, consumers fears and safety.	Poland	N/A	Scoping Review
			Stephens, J. et al.	Food delivery apps and the negative health impacts for Americans	United States of America	N/A	Editorial
			Wang, C. et al.	Hunger for Home Delivery: Cross-sectional analysis of the nutritional quality of complete menus on an online food delivery platform in Australia.	Australia	13,841 food items from 202 outlets	Cross-sectional Observational Study
Physical Activity and Sedentary Behaviors	Physical Activity; Sedentary Behaviors	physical activity, sedentary behavior, energy expenditure, factors affecting, COVID-19, isolation, leisure time, work from home (WFH)	Tremblay, M. S. et al.	Physiological and health implications of a sedentary lifestyle.	Canada	N/A	Narrative Review
			Füzéki, E. et al.	Physical activity During COVID-19 INDUCED lockdown: Recommendations.	Germany	N/A	Editorial
			Tison, G. H. et al.	Worldwide effect of COVID-19 on physical activity: A descriptive study.	187 countries	455,404	Longitudinal Observational Study
			Genin, P. M. et al.	Effect of a 5-month worksite physical activity program on tertiary employee’s overall health and fitness.	United States of America	95 employees	Longitudinal Observational Study
			Bourdas, D. I. et al.	Impact of COVID-19 lockdown on physical activity in a sample of Greek adults.	Greece	8495	Cross-sectional Observational Study
			Tan, S. et al.	Physical activity, Sedentary behavior, and Weight status of university students during the COVID-19 Lockdown: A Cross-National comparative study.	Malaysia	254 students	Cross-sectional Observational Study
			Constandt, B. et al.	Exercising in times OF Lockdown: An analysis of the impact of COVID-19 on levels and patterns of exercise among adults in Belgium.	Belgium	13,515	Cross-sectional Observational Study
Effects of Restrictive Measures towards Dietary Habits and Physical Activity	Dietary Habits; Physical Activity	diet, dietary habits, eating behavior, lifestyle, physical health, physical activity, sedentary behavior, pandemic, confinement, countries, changes, comparisons, quarantine, COVID-19	Shimpo, M. et al.	Shimpo, M. et al., Factors associated with dietary change since the outbreak of COVID-19 in Japan.	Japan	6000	Cross-sectional Observational Study
			Wang, X. et al.	Bidirectional influence of the COVID-19 pandemic lockdowns on health behaviors and quality of life among Chinese adults.	China	2289	Cross-sectional Observational Study
			Rodríguez-Pérez et al.	Changes in dietary behaviours during the COVID-19 outbreak confinement in the Spanish COVIDiet study	Spain	7514 participants	Cross-sectional Observational Study
			Papandreou, C. et al.	Comparing eating behaviours, and symptoms of depression and anxiety between Spain and Greece during the COVID -19 outbreak: Cross-sectional analysis of two different confinement strategies.	Spain, Greece	1841 total (1002 in Spain, 839 in Greece)	Cross-sectional Observational Study
			Sañudo, B. et al.	Objectively-assessed physical activity, sedentary behavior, smartphone use, and sleep patterns pre- and during-COVID-19 quarantine in young adults from Spain.	Spain	22 students	Longitudinal Observational Study

## 3. Result and Discussion

This section will discuss the outcome from the search strategy and study selection. The subsections after outlining the articles based on the applied strategy as in Table 1 would be the contents of discussion on the effects of the COVID-19 pandemic and restriction measures, followed by lessons learnt and policy recommendations.

Table 1 summarizes the articles used for main information on the effects of the COVID-19 pandemic and its restriction measures. The table is first divided into five themes (Dietary Behavior, Nutrition, Food Options, Physical Activity and Sedentary Behaviors, Effects of Restrictive Measures towards Dietary Habits and Physical Activity) before it is further divided into several subthemes. These subthemes are organized based on the content of the themes in the text review hence keywords used to strategically search through databases are based on the subthemes accordingly. There are 64 articles used for all nine subthemes with a variety of study designs that have been filtered and deemed applicable for the purpose of review. Most of the articles that used research methods were observational studies (43%) while most of the reviews used were narrative reviews (18%). Upon full-text review, 105 literatures were used.

### 3.1. Effects of the COVID-19 Pandemic and Restriction Measures

The effects of the COVID-19 pandemic and restriction measures are divided into five main themes which are “Dietary Behavior”, “Nutrition”, “Food Options”, “Physical Activity and Sedentary Behaviors”, and “Effects of Restrictive Measures towards Dietary Habits and Physical Activity”.

#### 3.1.1. Dietary Behavior

The sudden implementation of strict lockdown measures has caused a massive change in the lifestyles and the living environment of people around the world. Normal movement was restricted; people were asked to reduce social contacts and were required to quarantine in their own homes as a way to reduce the spread of COVID-19. This has led to a reduction in the purchase of fresh groceries such as fruits and vegetables as well as an uptick in the purchase of non-perishable goods such as canned foods [7]. For a large part, this was caused by the panic buying and stockpiling [8] among consumers especially after the restriction announcement. This accumulated to a snowball effect of less fresh food consumption, shifting the pattern of expenditure to food that would last longer—e.g.: pasta, canned tuna and dry soups among others [9]. The prolonged consumption of preserved and canned food over fresh food may lead to weight gain as well as an increased chance of contracting chronic non-communicable diseases [10] such as heart disease and diabetes. These possible secondary effects of the pandemic will only be noticeable many years from now; an outcome that could perhaps be avoided with an increased consumption of fruits and vegetables, which has been linked to a reduced risk of chronic diseases [11,12]. This is of particular significance especially in the time of the COVID-19 pandemic, as nutrition plays a vital part in modulating the body’s immune system—a response that is compromised in people suffering from long-term chronic diseases [13], which may lead to increased mortality.

The upsurge in the purchase of less fresh food could also be attributed to food insecurity and the instability of food supply chains [9] throughout the COVID-19 pandemic. As the virus ravaged communities, many governments implemented lockdown measures of varying degrees. Unfortunately, this meant that many food-processing plants and labor-intensive businesses were forced to close [14] with only essential businesses such as grocery stores and hospitals being allowed to open. This has interrupted the food supply chain, leading to disruptions that ranged from minor inconveniences to the formation of bottlenecks in supply chains [15]. Farmers and food producers that were reliant on exports, especially those dealing with perishable and specialty foods, suffered when border closures and travel restrictions were announced, with many incurring devastating losses [16]. In the earlier phases of the pandemic, some countries in their efforts to maintain the continuity of their resources and ensure food safety for their own population, imposed restrictions on imports of staple foods such as wheat. The reduction in the supply of these products, which remained to be in a sustained demand, directly influenced the prices to be constantly high [17,18]. This essentially restricted access to certain demographics of the population, further exacerbating food insecurity to further increase consumption of canned and preserved food as well as a reduction in fresh food intake [19].

However, some communities experienced an opposite trend, especially among food-secure households and high-income countries, in which a rise in consumption of fresh food was observed due to the sudden boom in home food procurement (HFP) activities. These HFP activities include pursuits that range from home gardens and foraging in the wild to fishing and hunting [20]. For some, home gardens are a way to reduce the stress and anxiety [21] that they faced during lockdowns but to others, home gardens were an essential financial need to reduce costs of grocery shopping [22], particularly in lower-income households. Besides that, people who engaged in home gardens and community gardens were more inclined to incorporate fresh produce into their daily diets [23] and be aware of the nutritional content of their food and were thus more inclined to make permanent positive changes to their lifestyles and diets [24]. In the long run, this will translate into a reduced risk of chronic diseases. In terms of the food supply chain, initiatives such as this may help to reduce food insecurity and provide an alternative to demographics that would otherwise not be able to access healthy food options due to geographical as well as financial limitations [25].

Another aspect in which restriction measures affected dietary behavior is via alcohol consumption. Some nations such as Germany, the United Kingdom and the United States of America reported an increase of 3% to 5% in alcohol consumption in 2020 compared to the previous year [26]. Lockdowns and restrictions enforced on social activities may have caused the sales of alcohol in bars and similar establishments to decrease sharply. However, it may have also caused people to shift their consumption of alcohol in the comfort of their homes. Online platforms such as Minibar and Drizzly in the United States observed an increase of sales—which soared past projected expectations during the pandemic [27]—as customers inclined to purchase bigger servings of alcoholic beverages [28]. The increase in alcohol consumption could, to some extent, be influenced by the increased psychological distress experienced by people during lockdown periods or ‘Shelter-In-Place’ orders. Stress has been shown to be a factor for chronic alcohol use and dependency. Prolonged alcohol use has the potential to permanently alter reward and stress pathways in our neural circuitry [29], resulting from the dysfunction of the hypothalamic–pituitary axis causing excessive stimulation, which affects emotional modulation. Such adaptations are far from healthy and may encourage alcohol consumption in response to stressful situations, leading to alcohol dependency and addiction. On the other end of the spectrum, during the short-term period following the implementation of restriction measures, alcohol use may be reduced among certain subsets of the population. The closure of establishments that traditionally sold these beverages such as bars, pubs and liquor stores may have contributed to this trend. This effect would mainly affect younger adults who live in college accommodation as their main avenue of consumption was bars or restaurants [30]. Abrupt closures would have meant that they had no alternative way to consume alcohol.

#### 3.1.2. Nutrition

The sudden change in the pattern of social behavior coupled with the intense uncertainty of the nature of the SARS-CoV-2 virus and its spread caused a lot of psychological distress especially in the beginning of the pandemic. Citizens of many countries have reported experiencing anxiety, hopelessness and boredom due to the imposed lockdown [31]. Unsurprisingly, many turned to carbohydrate-rich and fat-rich foods, which are infamously referred to as ‘comfort foods’, as a maladaptive way to self-soothe their emotions. Such classes of food are proven to be able to reduce stress due to the production of neurotransmitters such as serotonin and dopamine [32], which might be a possible explanation as to why ‘comfort eating’ spiked during the pandemic. These types of food are also classed as ‘hyper-palatable’ foods, which essentially results from the combination of certain ingredients such as fat, sugar, salt, and simple carbohydrates making it more appealing to our taste buds [33]. Research has also shown that the combination of fat and carbohydrate in food activates our brain reward circuitry at a much faster pace than fat or carbohydrates alone [34]. Constant stimulation of our reward system may lead to reduced activation of the physiological satiety mechanisms which promote over-indulgence in hyper-palatable ‘comfort food’. This poses a worrying health risk as these types of food are generally low in nutrients and may stimulate pro-inflammatory responses [35] in the human body. In the long term, such patterns of eating may cause malnutrition problems, generating more burden on already strained healthcare systems.

Nutrient-dense foods are key to preventing overeating and rapid visceral weight gain [36]. This is notably important as the aggregation of fat especially around the viscera has been distinctly linked to the development of a myriad of diseases such as diabetes mellitus due to increased insulin resistance, cardiovascular diseases and increased predisposition to certain types of cancers [37]. An increased amount of abdominal fat has also been associated with increased nosocomial infections as well as increased hospitalization and mortality rates among obese patients [38]. Recent studies have highlighted the existence of an association between obese patients and an increase in the severity of respiratory infections [39]. This could be due to the fact that adipose tissue can further induce a long-term low-grade inflammation [40], defined as heightened levels of pro-inflammatory cytokines such as TNF-alpha and reduced numbers of anti-inflammatory cytokines, partly because of its role as an endocrine organ that secretes bioactive substances. This chronic inflammation can reduce the capability of the body to eradicate the virus from its host, which might explain the increased mortality seen in obese or overweight COVID-19 patients.

The emergence of COVID-19 has shone a spotlight on the difference in nutrition and its corresponding changes in different diets, which include the Mediterranean diet, Nordic diet and intermittent fasting as a part of being healthy during the lockdown period. The Mediterranean diet is one that is rich in plant-based food such as fresh fruits and vegetables, legumes, nuts, seeds, and olive oil [41] and differs slightly to the Nordic diet that depends on rapeseed oil. The combination of healthy fats and a protein-rich diet in both diets is beneficial to the general population as it is rich in components that contain antioxidants, anti-inflammatory properties, and immunomodulatory properties, which include fiber, vitamins, minerals, polyphenols, flavonoids, and other micro-constituents [42]. These specific properties are even more pronounced when we discuss the pathophysiology of COVID-19 infections, which include cytokine storms and an increase in the activation of thrombotic effects [43]. Micronutrients such as polyphenols have been shown to hamper the process of apoptosis and cytotoxicity [44], thus regulating immunity as well as reducing inflammation [45]. Through this, polyphenols could positively influence a number of chronic diseases such as diabetes mellitus, which may predispose one to COVID-19 infections and a subsequently worse outcome [46] by reducing the levels of pro-inflammatory cytokines [47]. As such, practice of these diets could reduce the incidence of chronic diseases that may predispose patients to worse impacts of COVID-19 infection. There have been studies conducted to investigate the relationship between Mediterranean diet adherence and COVID-19 outcomes, most notably a Spanish study that observed a negative association between Mediterranean diet and COVID-19 cases across 17 regions in Spain and 23 other countries, after adjusting for factors such as physical activity and other determinants of well-being [48]. It may also help reduce the strain on our healthcare system as it has been linked to shorter hospital stays in the elderly population aged 65 and above as well as reducing the financial burden due to hospital bills in the long run [49]. The motives behind food consumption and purchases, and hence the adherence to the Mediterranean diet or any similar diets, could be influenced by the COVID-19 pandemic and have thus varied before and during the pandemic. This was observed by a study in Iran, which showed that sensory appeal and price were some of the more crucial factors behind food consumption in the pre-pandemic period, while health as well as weight management were noticeably more important factors during the COVID-19 pandemic period [50].

Intermittent fasting (IF) is a diet regimen that switches between fasting and eating on a regular schedule [51]. There are many variations to it in terms of hours. For example, some may opt to fast for 16 h with an 8-h eating window (16:8) while others may choose to increase their fasting times to 20 h with a 4-h eating window (20:4) [51]. In terms of its application in the current pandemic, it has been indicated that increased cellular glucose metabolism mediated by an increase in hexosamine biosynthesis [52] could contribute to the exacerbation of COVID-19. Once this metabolic pathway is triggered, cytokine storms can be induced through upregulation of IR-5 [52] particularly during viral infections, a process that is linked to worse COVID-19 outcomes [53]. Thus, impeding the normal pathway of glucose metabolism and its related pathways may help to reduce cytokine storms. Besides that, elevated glucose levels have been linked to an increase in the level of glycolysis that promotes replication of SARS-CoV-2 and pro-inflammatory cytokine [54]. Hence IF is suggested to be beneficial for metabolic health and weight loss [55]. IF also exerted a positive effect on insulin sensitivity, oxidative stress and blood pressure [56], which may improve the outcome and severity of COVID-19 infection in the population. IF has been demonstrated to reduce adipose tissue composition, which has been noted to be a SARS-CoV-2 reservoir due to the high expression of angiotensin converting enzyme 2 (ACE2), which has been shown to facilitate viral entry [57]. These results raise the prospects of using IF as a potential preventive measure to lower adverse outcomes following COVID-19 infection. However, more data are needed to support this approach should it be applied to the general population.

#### 3.1.3. Food Options and Food Delivery Usage

Even before the COVID-19 pandemic and its accompanying restriction measures, food delivery apps were experiencing a rapid growth in the last decade—as a part of a trend that demonstrated customers’ shift to e-commerce and other similar means of commerce. This was caused by the interaction of various factors such as an increase in income and access to the Internet, safe e-payment alternatives, longer commuting and waiting times for eating outside as well as a better grasp on the mechanics of e-commerce [58]. Restriction and lockdown measures were merely an accelerant to this pattern of obtaining food. Generally, food delivery apps (FDA) can be classified into two main categories—Restaurant-to-Consumer and Platform-to-Consumer delivery [58]. For the former, the restaurant itself will receive orders and dispatch delivery riders to fulfill their orders. Thus, third parties are, more often than not, not included in this business model. Some of the more well-known franchises that currently adopt this model include McDonald’s™, KFC™, and Domino’s Pizza™—each with its own delivery applications. The second business model would be the platform-to-consumer delivery model, which the majority of food delivery apps utilize [58]. This is where restaurants and small businesses advertise their food products on platforms that will then employ drivers or ‘riders’ to deliver their orders. Examples of these platforms include GrabFood™ in Southeast Asia, Swiggy™ and Zomato™ in India, Just Eat™ and Deliveroo™ in the United Kingdom as well as Grubhub™, Doordash™, and Uber Eats™ in the United States.

In the early phases of the COVID-19 pandemic, restriction measures were implemented to actively discourage people congregating in places that lacked proper ventilation and crowded facilities such as supermarkets, for the purpose of breaking the COVID-19 transmission chain. This, coupled with the fact that the virus was far more dangerous than existing respiratory infections, motivated many people to comply with stay-home orders. Although beneficial in reducing infections, it changed the daily routine of many sections of the community. With the suspension of commuting to work and the implementation of work from home (WFH), many people found themselves reverting to food and grocery delivery to avoid contracting or spreading the disease [59]. This directly created a shift in the demand for store-to-door delivery services that propelled the sudden growth of FDAs. The reasoning behind the swift change of this new food obtaining pattern was twofold—people were prepared to pay higher amounts of money, which included delivery fees and tax for the ease and comfort that was afforded by FDAs, while drivers and independent contractors were motivated to work for less as unemployment rates shot up [59].

Many also looked to break the monotony of staying at home by ordering food from various cuisines. Because dining out was not an option due to restrictions on social activities, FDAs provided the next best alternative—delivery to the doorstep of their house. This not only provided ease and comfort but also the reassurance of the hygiene and sanitary standards of the food prepared as well as the environment it was prepared in [60]. FDA owners realized the importance of this reassurance in driving up sales and began implementing changes such as contactless delivery and body temperature checks at every stage of preparation and delivery. An example of this would be McDonald’s™ Malaysia’s practice of sealing each delivery bag with a sticker stating the temperature of their workers who handled the foods, which included their delivery riders [61]. Other more locally well-known platforms such as Foodpanda™ and Grab™ in Malaysia started offering contactless delivery that was enhanced with the introduction of payment methods besides the traditional cash-on-delivery (COD).

However, the ease and convenience of FDAs bring with it a whole set of problems and future complications. Eating out, or in this case, ordering in, has been positively associated with a higher BMI [62]. A survey of over 2900 consumers in the United States showed that in the past 90 days [63], 41% of their respondents reported having used a platform-to-consumer-based FDA. Of those who had used FDAs, 52% reported ordering food upwards of three times. Although some may argue that FDAs have created an avenue for the consumption of healthier and more nutritious food, most of the existing research says otherwise. One study showed that food that did not meet the Five Food Groups (FFG) requirement was more than two times more likely to be marketed as more popular than its counterparts [64]. Similarly, non-FFG compliant food was almost seven times more likely to be marketed as a package, which influenced people to consume more. It follows the marketing psychology of a supermarket [65] displaying the more expensive items at eye level to sway customer consumption in their favor. From a nutritional standpoint, non-FFG food is calorie-dense and high in salt, sugar or oil, as well as low in fiber. This poses a threat to public general health, especially in the time of the COVID-19 pandemic, as sustained consumption of such classes of food will only serve to exacerbate the existing obesity epidemic in the long run. In the more immediate time frame, poor nutrition has been linked to increased mortality and increased ICU admissions when infected with SARS-CoV-2 [39].

#### 3.1.4. Physical Activity and Sedentary Behaviors

The spread of the COVID-19 virus with its current state of transmission has urged public health to heavily depend on social and behavioral change strategies beyond strict hygienic rules. Some of the strategies include isolation, social distancing and quarantine, as per World Health Organization recommendations [66]. The success of these strategies occurs with the rapid shifting to lifestyle changes such as work from home (WFH) that has increased steeply and persistently for offices and corporates after the outbreak. These measures have limited the amount of physical activity, as it is fair to assume that WFH implementations will result in an expected reduction of transportation activities. Besides having to deal with work activities infiltrating the comfort of our homes, sports and fitness clubs are also closed—ideally to fulfill the social distancing strategy, hence this potentially reduces the level of physical activity.

Decreased level of physical activity is also attributed to increased sedentary behaviors during the lockdown pertaining to working from home [67]. Sedentary behaviors are any behaviors when awake with an energy expenditure ≤1.5 of the metabolic equivalent (METs) in a sitting or lying posture [67]. These two entities are entirely independent because an individual may be highly sedentary due to their work requiring a large amount of focus on the computer hence high sitting time, but they may or not be a physically active individual; it depends on whether they meet their physical activity recommendations based on their age outside of work [68]. The World Health Organization (WHO) outlined that adults aged 18–64 years are recommended to perform at least 150 min of moderate-intensity, 75 min of vigorous-intensity physical activity, or a balanced combination of moderate-to-vigorous physical activity (MVPA) per week as regular physical activity is proven to prevent non-communicable diseases such as diabetes in addition to improving mental well-being. Any energy expenditure lower than the recommendations would define an individual as inactive, hence the synergistic effect of a sedentary lifestyle and being physically inactive can potentially increase the mortality rate. Staying at home due to COVID-19 is a sedentary measure that reduces physical activity. There is evidence reporting an overall negative change in physical activity among adults during the ongoing COVID-19 outbreak with an excess of leisure time activities in the lockdown environment [69]. The active promotion of online activities adhering to rapid lifestyle shift as a COVID-19 preventive measure have also been linked to an expected high prevalence rate of physical inactivity from certain subgroups in the population, such as students who are highly prone to screen exposure that would be enough to postulate extended periods of insufficient physical activity participation turning into sedentary behaviors [70].

Physical and social environmental factors influencing physical activity participation are based on availability, utilization and ease of access. It is important to note that the level of physical activity ultimately depends on every individual’s initiative. The plausible reasons are due to the unavailability to exercise with friends and lack of interest to continuously participate in physical activities due to the obvious loss of the competitive element during exercising from the lifestyle shift [71]. Despite the dramatic reduction of physical activity from the closure of educational institutions, there are populations of Malaysian university students who are notably more physically active during the lockdown period, demonstrating better engagement in physical activity, even though current findings showed less compliance to WHO recommendations [72]. The social ecological framework shapes physical activity behaviors and a simple socioeconomic background such as rural or urban living of an individual can be a causal factor associated with physical inactivity and sedentary behaviors, especially in this drastic era of lifestyle changes due to the COVID-19 outbreak [73].

#### 3.1.5. Effects of Restrictive Measures towards Dietary Habits and Physical Activity

Plenty of research has been conducted since the COVID-19 pandemic began, particularly on dietary and eating habit changes. Based on an online cross-sectional questionnaire survey conducted at one time point in November 2020 when the Japanese government issued stay-at-home requests, only 8.2% adults out of the 6000 respondents experienced an unhealthier change from their initial dietary habits as they live alone with higher stress levels from the lack of social support, while 71.6% reported unchanged diets [74]. The unchanged dietary habit was also noted from 34 provinces across China with 71.4% of the participants noting no changes in their appetite [75]. Interestingly, data from Spain showed a high cohesion to the healthy Mediterranean diet with 63.7% declared having not eaten more during the first 3 weeks of confinement period [76]. Unlike Spain, Greece had a lower MedDiet score as they adhere less to the diet, showed a greater weight gain and the participants binge ate more between meals [77]. As all of the aforementioned countries have different geopolitical and economical statuses that create their own respective impact on dietary and eating habits during COVID-19 pandemic implemented restrictions, the similarity that unites the studies regardless of the participants’ sociodemographic profiles was the reduced level of physical activity. Despite the large portion of participants reporting no changes in dietary habits, adults in China did not perform moderate-intensity (40%) and vigorous-intensity (55%) physical activities [75]. A study among young adults in Spain, which objectively measured and compared the physical activity before and during lockdown period using the mean steps taken per day, had shown a significant decrease of 67.7% during the lockdown period [78]. Data from Greece similarly showed an apparent increase in inactivity by 40.6% with a dramatic decrease of 84.7% in competition sports held during the lockdown period [71]. Japan, which had an initial relatively low infection rate and non-instituted lockdown, still exhibited a decrease in overall step count with a 15% reduction over 24 days, from the data obtained on smartphone algorithms [69]. To date, different instrumentations have been used to study the effect of COVID-19 restrictive measures on both physical activity levels and dietary behaviors while taking into account other possible individualistic factors, but the immediate impact observed worldwide is the reduction in physical activity. As dietary behaviors differ with different populations, restrictive measures have impacted our lifestyle completely by reducing as many movements as possible, including through converting homes to workplace settings that directly contribute to an instant decrease in physical activity.

### 3.2. Recommendations

The recommendations in the following paragraphs will describe the lessons learnt and the consequential policy recommendations; it is also another subsection that acknowledges the shift of COVID-19 pandemic to an endemic phase.

#### 3.2.1. The Lessons Learnt and Policy Recommendations

The lessons learnt from the past years will be instrumental in the development of effective public policies in the future-some of which may have helped in mitigating the harmful fallout that the world is witnessing during the pandemic and the subsequent implementation of restriction measures.

High anxiety levels among the citizens should be mitigated, thus governmental agencies should ensure that information related to COVID-19 should be communicated to all levels of society comprehensively. This would include ensuring that this information is appropriately presented to the public to ensure maximum understanding. Additional data on preventative measures should also be frequently updated using various platforms such as social and print media to reduce fear and reassure the general public of the scientific basis behind such actions. This will allow the public to easily access health information, particularly related to the COVID-19 as they are regularly exposed to and consume media from these sources [79]. These spanned from information provided by governmental organizations to information obtained from mass media and peer-educators.

The rapid development of Internet services worldwide has allowed citizens to easily access healthcare services. This indeed should be an integral part of patient care. In a world that has adapted physical distancing as a new normal, online consultations with qualified medical practitioners, particularly mental health care providers, are critical at this juncture [80]. According to a WHO survey [81] which was conducted across 130 countries, the pandemic has introduced major disruptions to mental health services in 93% of nations. In a time when more people are reporting symptoms of mental health disorders such as anxiety and depression, it is vital that governmental agencies, as well as private healthcare providers, understand the importance of psychological support especially to those who are in crisis or have limited access to healthcare. Services such as teleconsultation and therapy services including ”Dialectical Behavior Therapy” (DBT) and “Cognitive Behavior Therapy” (CBT) can be carried out through these channels [82]. The E-healthcare services have been shown to reduce the number of clinic visits and further help to curb transmission of the virus. Simultaneously, it also reduces the medical expenditure of patients [83], expanding access to healthcare across a wider demographic of the population.

While the COVID-19 pandemic has caught governments across the world off guard, it offers a reset in terms of how government policy is implemented during a pandemic. The fear of being evicted, the inability to purchase foodstuffs, and the reduction in income flow has without a doubt contributed to a psychological scar for many individuals worldwide. With climate change threatening to unleash a plethora of new viruses, political leaders need to move away from the romanticisation of the deficit during a time of national crisis as after all, the aim of a government is to maximize social welfare and not profit. The introduction of various stimulus packages can help citizens of affected countries during the pandemic. As such, moving forward governments should aim to be proactive by drawing up stimulus packages that cover the lower-income groups. The government should offer subsidised RT-PCR testing and free facial coverings for individuals. The benefit of such measures will be far-reaching to even the most vulnerable populations who probably do not have access to comprehensive medical insurance. Relying on testing only when symptomatic and in emergency care settings is inefficient in the long run as more and more members of marginalized communities will fall through the cracks that build in our healthcare system. 

Confinement imposed by governments calls for a necessary elaboration of effective strategies to ensure already active individuals sustain their physical activity level while also prompting inactive people to pick up their activity. These strategies that adhere to the restrictions would inevitably aid in combating sedentariness which is obviously favoured by many since the introduction of work-from-home (WFH) [84]. By taking advantage of the increased teleworking hours due to WFH and use of social media, the government should increase efforts to encourage diverse physical activity research. The increased number of publications, especially in this COVID-19 pandemic that involved a drastic change in the human lifestyle had presented evidence to prove the strong association between declining physical activity and increased sedentary behaviors, but these publications were mostly limited to observational studies [85]. Now as we move towards living with the COVID-19 in the community, it is important to strengthen the research capacity by doing studies with a focus on objective measures that would help in identifying factors influencing effective interventions with the primary goal of curbing continuous decline in physical activity level and incline of sedentariness. The active usage of the official public health social media profiles ever since the pandemic started has been very impactful. Designing a standardised toolkit for developing policies to incorporate physical activity in workplace settings that can be applicable for WFH as well could encourage and motivate workers to engage in physical activity. The government should also increase collaboration efforts with non-government organizations and ministries so that resources can be optimised maximally for bigger engagement with the society and enlarge evidence-based methods for physical activity promotion [86]. 

With regard to nutrition and food choices, there should be more government intervention in setting price ceilings for healthier options. As it stands, healthier and more sustainable food is priced higher than mass-farmed and preserved food which may serve as a barrier for lower-income families to choose healthier options. Therefore, the government should heavily subsidize staple foods such as rice, wheat, bread, vegetables and eggs so they can be purchased by all. A food pyramid of eating healthy should be included in the campaigns to educate consumers about making healthier food choices and empowering them by providing as much information as possible. Some countries have already formulated dietary guidelines that serve as a starting point for people wanting to make the change. In Scandinavian countries such as Norway and Denmark, a ‘keyhole’ symbol that is displayed on the front packaging of certain edible products has been adopted to symbolise more nutritious food [87]. Similarly, Australia and New Zealand utilize a star rating system [88] to inform consumers of the nutritive value of products. A higher rating would be given to food that was more nutritionally balanced [88] and thus more beneficial to the population. Ratings like these would be beneficial if they were adopted by more countries. Alternatively, these ratings could be standardized to provide more ease to consumers worldwide to keep track of their nutritional intake.

In terms of food delivery, governments could legislate laws and regulations that require platform providers to clearly show the nutritional breakdown of food in terms of macronutrients, micronutrients, and minerals. This would empower patrons to consciously try to make the switch to eating healthier. Currently, many platforms vaguely label their options with words such as ‘gluten free’, ‘oil free’, ‘reduced sugar’, and others without any oversight. Thus, there is no standardization on what exactly these terms mean which may be misleading for consumers. Besides that, governments could opt to increase taxes on food high in sugar, oil, sodium, and others in an effort to deter the widespread consumption of these classes of food [89]. However, these efforts would need to be coupled with subsidization programmes for fresh food such as fruits and vegetables as a stand-alone taxation policy may be regressive in nature. This is because ‘junk food’, that is unhealthy, is usually sold at a cheaper price and may be the only other alternative to lower-income families [90]. Taxing these classes of food without offering monetary aid or other forms of subsidy would only serve to further disadvantage them. Many countries worldwide have adopted ‘sugar taxes’ or ‘junk food taxes’ including the United States of America [91] and Hungary [91]. Although these measures have been shown to reduce consumption of those food groups, there has not been a simultaneous effort to increase the accessibility and affordability of fresh food. This could easily be achieved by channeling the increased national income from the taxes into health programmes and subsidies that serve to influence citizen’s dietary habits for the better. No single approach will be able to tackle the complexities of people’s food choices. However, governments should actively engage with stakeholders involved to find a combination of policies that will promote healthy living with the ultimate goal of a healthy nation. 

As countries around the world start to embrace the approach of treating COVID-19 as an endemic disease [92], the importance of academic institutions and their role in promoting health behaviors and management is increasingly being highlighted. Throughout the COVID-19 pandemic, many countries have taken the pre-emptive measure of closing schools taking into account children’s major potential of spreading and contracting respiratory viruses due to their close interaction in learning institutions [93]. While there is a myriad of resources from organizations such as WHO and the respective national ministry of health [94,95], these materials are not readily disseminated to children and teenagers. There is an obvious lack of age-appropriate materials, especially in a local context, that are available and easily digestible for specific levels of the population. For example, for younger children who might not be as aware of the danger of COVID-19 infections, effective infographics that are both interesting and informative are needed such as concepts of social distancing and hand hygiene. Public health issues have been discussed in schools to educate not only students but their families as well [96]. Subjects like Physical Education have long been used to deliver messages regarding a healthier lifestyle like obesity and smoking reduction [97]. It is not a far reach to adapt this form of messaging to target school-going children and their families to help foster better knowledge on COVID-19 and ways to reduce transmission as well as educating them about the significance of a healthy lifestyle—adequate physical activity, nutritious food as well as avoidance of smoking and excessive alcohol consumption—in reducing the adverse outcomes following COVID-19 infection [98]. Schools and other learning institutions can play an integral role in disease prevention by communicating and capturing the attention of students to aid the effective sharing of science-based measures and information related to breaking the chain of transmission in an effort to equip them with sufficient knowledge and self-efficacy to adopt preventive behaviours such as physical distancing and wearing masks [99]. However, incorporating these aspects into the current education curriculum and ensuring that the information is accurate and up-to-date, a close cooperation between various stakeholders is needed.

#### 3.2.2. COVID-19 Moving towards Endemic

Going into two years after the world was made known of the novel SARS-CoV-2 virus, the global COVID-19 prevention strategies would have to be redefined to counteract the detrimental effects the pandemic has impacted on us. From individual behavioral changes to the nationwide economic losses, SARS-CoV-2 virus has become not just pathogenic to human health, but has also proven to be equally “pathogenic” to human lifestyle, thus this virus threat has to be managed promptly and holistically to regain a better quality of life. Terms such as ‘eradication’, ‘elimination’, and ‘COVID Zero’ are current trends of the COVID-19 strategies proposed towards ending the current state of SARS-CoV-2 community infection, which is now gradually considered as an epidemic in many nations. Ultimately, all the terms are defined differently according to respective countries; United States health personnel use ‘elimination’ as an action-oriented definition of maximizing “action to control SARS-CoV-2 and stop community transmission as quickly as possible”, which is also an approach similarly favored by the New Zealand government [100]. ’COVID Zero’ is the term describing the policy of eradicating COVID-19 transmission to the extent of achieving zero reported cases in the community. Nonetheless, countries with the initial plan to maintain COVID Zero by both blocking foreign arrivals and shutting state borders, such as Australia, have rearranged their strategy upon the identification and transmission of the Delta variant, which caused the Australian authorities to conclude that COVID Zero is no longer achievable [101]. Hence, Australia re-strategized with a heavy reliance towards vaccination, with the Groundhog Day ending when 70–80% of the citizens are fully vaccinated [101]. With a similar approach, England has announced the reopening of their borders to fully vaccinated travelers from the United States and Europe without needing to quarantine starting from early August 2021, as England managed to achieve 86.7% full vaccination of their adult population aged 16 and above [102]. As of September 2021, Singapore observed the reality of endemic COVID-19 as seven-day daily average cases were higher than their previous waves of pandemic, while it remained to have low COVID-19 deaths, which was mostly contributed to by its high vaccination coverage of over 80%. Malaysia, being the latest country in Southeast Asia (after Singapore, Thailand and Indonesia) to relax travel restrictions by lifting the ban on interstate travels on 11 October 2021 upon achieving 90% full vaccination rate among its adult population, has transitioned to an endemic phase by the end of October [103]. Therefore, it is worth noting that the direction towards the endemic phase for most countries at this point in time is by relaxing travel restrictions, which would positively impact several aspects such as psychosocial and emotional well-being through the reunion of separated loved ones, and boosting the sagging tourism and transportation industries as borders can be crossed, and visitations can be permitted. To achieve this form of travel freedom is by aiming towards achieving herd immunity through high vaccination rates, so that there are more fully vaccinated adults in the community, while maintaining current COVID-19 transmission prevention standard procedures. Enforcing COVID-19 vaccination across as many groups of people in the population based on respective countries’ policies will make the process of regaining a healthier lifestyle with the needed adaptation to COVID-19 standard procedures possible [104]. To date, vaccination is the only known approach which can be effectively employed to curb this COVID-19 pandemic in such a large scale, while the health community is eagerly waiting for more interventions such as specific antiviral or SARS-CoV-2 monoclonal antibodies to be adequately tested and made available [105].

## 4. Conclusions

This scoping review highlights the varying effects of the COVID-19 pandemic and restriction measures towards aspects of human lifestyle. These include dietary behavior and nutrition, food options and food delivery usage, physical activity and sedentary behaviors. The influence of social groups and economic standings on how these effects were perceived was underlined in this review. The shift from the novel pandemic to an endemic phase was significant to be highlighted in this literature to present the re-evaluation of COVID-19 prevention strategies by many governments at the time of writing, which is fitting to be discussed, as the world needs to continue living rather than bearing the damaging long-term effects that both the pandemic and restriction measures would continue to impact on humankind if no advancement is made.

To summarize, in an effort to halt the transmission of this virus, restriction measures were the preferred method for many countries. However, as we have discussed above, these came with their own set of problems which need to be addressed by governments and relevant stakeholders worldwide. In that sense, this review presents relevant literature to enable a more comprehensive understanding of the effects and lessons learnt from this pandemic—a tool that would be particularly useful for governments to tailor more effective responses for future pandemics. The long-term effects of the COVID-19 pandemic and its restriction measures are mutually exclusive in various aspects. Further research and reviews should focus on trying to understand the driving force behind these social inequalities and how we may work towards dampening the speed at which we experience the negative effects of living in a pandemic and a more restrictive and cautious environment.
